# Functional Nucleic‐Acid‐Decorated Spherical Nanoparticles: Preparation Strategies and Current Applications in Cancer Therapy

**DOI:** 10.1002/smsc.202000056

**Published:** 2021-02-09

**Authors:** Min Zhu, Shan Wang

**Affiliations:** ^1^ Department of Pharmaceutical Engineering College of Chemistry and Chemical Engineering Central South University No. 932 South Lushan Rd Changsha Hunan 410083 P. R. China

**Keywords:** cancer therapy, controlled drug release, functional nucleic acids, nucleic acids linking strategies, spherical nanoparticles, targeted drug delivery

## Abstract

Functional nucleic acids (FNAs) have drawn widespread attention in the construction of functional nanomaterials for biomedical applications due to their inherent biological functions and sequence programmability, as well as high thermal stability and easy chemical modification. FNA‐decorated spherical nanoparticles (FSNPs) are composed of a metal/metal‐free spherical core and a radially oriented FNA shell. Attracted by their unique capabilities, such as resistance to nuclease degradation and capability of crossing the blood–brain barrier, FSNPs as smart nanomaterials for cancer therapy are reviewed. The preparation strategies of FSNPs are first summarized, and the applications of responsive linkers in stimuli‐responsive drug release are introduced. The FSNPs are categorized into aptamer‐, i‐motif‐, DNAzyme‐, antisense oligonucleotide‐, and CpG oligodeoxynucleotide‐decorated SNPs. Their applications in cancer therapy include tumor‐targeting drug delivery and controllable releasing of drugs, overcoming physiological or pathological obstacles such as blood–brain barrier and interstitial transport barriers, as well as a reversal of resistance to chemotherapy and antitumor immune response activation. The remaining challenges and future directions of FSNPs are also discussed and proposed.

## Graphical abstract

The preparation strategies of functional nucleic acid (FNA)‐decorated spherical nanoparticles (SNPs) are summarized with the focus on the current applications of aptamer‐, i‐motif‐, DNAzyme‐, antisense oligonucleotide (ASO)‐, and CpG oligodeoxynucleotide (ODN)‐decorated SNPs in cancer therapy, involved in targeted drug delivery, controlled drug release, gene regulation, and antitumor immune response activation.

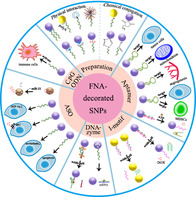

## 1 Introduction

With the rapid advance of nanotechnology, versatile nanoplatforms for drug delivery in cancer therapy have been increasingly developed.^[^ ^1^ ^]^ Among them, spherical nanoparticles (SNPs), the shape of which is common in nature, including metal (e.g., gold,^[^ ^2^ ^]^ iron oxide,^[^ ^3^ ^]^ etc.) and metal‐free nanoparticles (e.g., mesoporous silica,^[^ ^4^ ^]^ liposome,^[^ ^5^ ^]^ micelle,^[^ ^6^ ^]^ etc.), are popularly favored as drug‐delivery vehicles, and even as an adjuvant of therapy. Metal SNPs can carry drugs onto their surfaces via covalent or noncovalent approaches,^[^ ^7^ ^]^ and some of them also exhibit antitumor activity and imaging capability due to their unique optical and magnetic properties,^[^ ^8^ ^]^ which might make them prospective theranostic nanoplatforms. Metal‐free SNPs exhibit various properties such as biocompatibility, biodegradability, and chemical modification, as well as a high potential for multidrug delivery.^[^ ^9^ ^]^ However, the use of these SNPs has been dampened by several problems, including lack of active targeting capability toward the tumor, uncontrollable release of drugs, poor penetration across the blood–brain barrier (BBB), or weak permeability to interstitial transport barriers caused by the tumor‐associated fibroblasts and dense extracellular matrix in solid tumor.^[^ ^10^ ^]^ Fortunately, these obstacles of SNPs can be overcome after being decorated by various functional molecules, including small molecules (e.g., folic acid) and macromolecules (e.g., proteins and nucleic acids).^[^ ^11^ ^]^

Nucleic acids, including DNA and RNA, are key information‐carrying biological molecules essential for life. A number of unique features, such as high thermal stability, easy chemical modification, and facile surface immobilization, make nucleic acids extremely promising in the modification of SNPs. In particular, the specific functional nucleic acids (FNAs), which exhibit special recognition,^[^ ^12^ ^]^ responsiveness,^[^ ^13^ ^]^ catalysis,^[^ ^14^ ^]^ or even therapeutic potential, have attracted increasing attention.^[^ ^15^ ^]^ Actually, it is a “win‐win”. On the one hand, FNAs endow SNPs capabilities for targeting tumor‐associated molecules and controlling drug release on demand. On the other hand, the dense and oriented distribution of FNAs on the surface of SNPs (namely spherical nucleic acids, SNAs) can resist nuclease degradation toward FNAs,^[^ ^16^ ^]^ especially for therapeutic nucleic acids, which are responsible for overcoming multidrug resistance, activating the immune response, etc. Furthermore, with the special 3D structure, the SNAs can bind more strongly to class A scavenger receptors, which are highly expressed on endothelial cells that make up the BBB, resulting in the high penetration capability toward BBB.^[^ ^17^ ^]^

In this review, we focus on the preparation strategies and current cancer therapy applications of FNA‐modified spherical nanoparticles (FSNPs), composed of a FNA shell, a spherical metal/metal‐free core, and the linker. First, we will summarize linking strategies of nucleic acids (including FNAs) to SNPs involved in physical interaction and chemical conjugation. Subsequently, we will elaborate on the characteristics of typical types of FNAs and the related applications of FSNPs in cancer therapy. Finally, the current challenges and future perspectives of FSNPs will be discussed.

## 2 The linking Strategies of Nucleic Acids (Including FNAs) to SNPs

Compared with the SNPs, the compositions of nucleic acids (A, T/U, G, C) are relatively invariant. Thus, the linking strategies of nucleic acids (including FNAs) to SNPs are mainly dependent on the types of SNPs. In 1996, Mirkin et al. first reported nucleic acids could link with gold nanoparticles (AuNPs) via strong Au‐S chemistry.^[^ ^18^ ^]^ After that, various popular SNPs, including silver nanoparticles (AgNPs),^[^ ^19^ ^]^ micelles,^[^ ^20^ ^]^ superparamagnetic iron oxide nanoparticles (SPIONs),^[^ ^21^ ^]^ mesoporous silica nanoparticles (MSNs),^[^ ^22^ ^]^ liposomes,^[^ ^23^ ^]^ proteins,^[^ ^24^ ^]^ and extracellular vesicles (EVs),^[^ ^25^ ^]^ were successfully linked with nucleic acids.

There are two main linking strategies to prepare FSNPs: the first strategy (strategy 1) is that the modified nucleic acids directly link with the preformed SNPs, which is suitable for all types of core compositions; the second strategy (strategy 2) is that the modified nucleic acids are first conjugated with hydrophobic or amphiphilic ligands and then self‐assembly to form FSNPs, which just applies to liposome or micelle. In strategy 1, due to the diversity of composition and surface functionalization of SNPs, nucleic acids should be modified with the corresponding ligands, allowing for the attachment onto the surface of SNPs through physical interaction or chemical conjugation, whereas chemical conjugation is commonly used in strategy 2 for the formation of amphiphilic nucleic‐acid conjugates. As shown in **Table** **1**, we summarize the linking methods of the modified nucleic acids to SNPs from physical interaction and chemical conjugation aspects. Herein, physical interactions (including hydrophobic interaction, electrostatic interaction, desthiobiotin–avidin interaction, etc.) and chemical conjugations (including alkynyl–azide cycloaddition reaction, amine bond, thiol bond, etc.) are both involved in irresponsiveness and responsiveness.

**Table 1:** The linking strategies of nucleic acids to SNPs

Linking strategies	Linking methods	Nucleic acid modification	Coupling groups	Spherical nanoparticles	Ref.
Physical interaction	Hydrophobic interaction	Cholesterol	–	EVs Liposomes	[26a] [26b]
		DSPE‐PEG_2000_	–	Liposomes	[27]
		Diacyllipid‐PEG	–	EVs	[28]
		DPPE	–	Micelles	[29]
		α‐tocopherol	–	Liposomes	[23]
		β‐CD	BM	MSNs	[36]
		Azobenzene	β‐CD	AuNPs	[38]
	Electrostatic interaction	Chitosan	–	Liposomes	[40]
		Hyaluronic acid	–	Hybrids	[41]
		KALA	–	Proteins	[42]
	Desthiobiotin–avidin interaction	Desthiobiotin	Avidin	MSNs	[46]
	Other physical interactions	Poly(A)	–	AuNPs	[47]
		BSA	–	Micelles	[49]
Chemical conjugation	Bioorthogonal reactions	Alkynyl	Azide	MSNs	[53]
		DBCO		Micelles Liposomes	[55] [31]
	Amide bond	Amine	COOH	Liposomes	[56]
Chemical conjugation	Amide bond	Amine	COOH	Micelles	[57a]
				SPIONs	[57b]
				proteins	[57c]
			PDA	MSNs	[59]
			Succinic anhydride	–	[60]
			AC		[61]
	Solid‐phase phosphoramidite chemistry	–	Phosphora‐midite	Micelles	[63]
	Thioether bond	Thiol	Maleimide	Liposomes	[65]
				Micelles	[66]
				MSNs	[67]
	Other chemical conjugations	Thiol	–	AgNPs	[68b]
				AuNPs	[71]

### 2.1 Physical Interaction

#### 2.1.1 Hydrophobic Interaction

Hydrophobic interaction occurs because these molecules lacking affinity for water have a tendency to move closer together. Nucleic acids can be tethered onto the surface of EVs, liposomes, and micelles using hydrophobic lipids as bridges, which can insert into the hydrophobic lipid‐bilayer of EVs and liposomes or the hydrophobic core of micelles. Cholesterol (Chol), as an essential constituent of the cell membrane, was favorably used to anchor nucleic acids onto the surface of EVs or liposomes.^[^ ^26^ ^]^ Other hydrophobic lipids, including 1,2‐distearoyl‐*sn*‐glycero‐3‐phosphoethanolamine‐*N*‐[methoxy(polyethylene glycol)‐2000] (DSPE‐PEG_2000_), diacyllipid‐PEG, and 1,2‐bis(diphenylphosphino)ethane (DPPE) can assist nucleic acids to be anchored on the surface of liposomes,^[^ ^27^ ^]^ EVs,^[^ ^28^ ^]^ and micelles,^[^ ^29^ ^]^ respectively. Moreover, the difference in affinity of these anchors to the core nanoparticles can affect the stability and cellular uptake of DNA‐functionalized SNPs. For example, Meckes et al. prepared two types of liposomal SNAs with different anchors (i.e., Chol and DPPE) and found that the DPPE‐tailed liposomal SNAs showed a significantly greater DNA shell density and half‐life than the Chol‐tailed ones. The enhanced DNA shell density and stability of the DPPE‐tail liposomal SNAs, stemming from the higher affinity of DPPE than Chol to liposomes, further facilitated cellular uptake of liposomal SNAs through scavenger receptor‐mediated endocytosis.^[^ ^30^ ^]^ Furthermore, Ferrer et al. demonstrated that the Chol‐tailed liposomal SNAs showed high DNA accumulation in the lungs, whereas the DPPE‐tailed liposomal SNAs exhibited high DNA trafficking to the kidneys, and both of them displayed high accumulation in the spleen.^[^ ^31^ ^]^ The study indicates that the biodistribution of DNA‐functionalized SNPs is concerned with the type of anchor, which also should be considered when designing FSNPs for targeted cancer treatment.

The lipid‐soluble α‐tocopherol, the most active and effective form of vitamin E, is widely distributed in nature. The liposome was surface‐decorated by α‐tocopherol‐terminated nucleic acids through α‐tocopherol embedment in its hydrophobic region.^[^ ^23^ ^]^ Unfortunately, α‐tocopherol is very sensitive to oxygen and easy to be oxidized especially in alkaline solutions.^[^ ^32^ ^]^

In addition, the hydrophobic interaction of host and guest molecules can be an appropriate bridge as nucleic acids and SNPs. The nucleic acids are first modified with the host or guest molecules and then link with the guest or host molecules on the surface of SNPs. Host molecules exhibit binding sites that can specifically recognize an analyte “guest” to form the host–guest complex. Also host–guest interaction is just similar to the complementarity of a lock (host molecule) and key (guest molecule). β‐cyclodextrin (β‐CD) is a natural cyclic oligosaccharide consisting of seven D‐glucopyranose units and often shows a truncated cone‐shaped structure of a hydrophobic inner cavity and a hydrophilic outer surface.^[^ ^33^ ^]^ Thus, β‐CD has been used as a popular host molecule that can be inserted by several hydrophobic guest molecules. Among them, some are capable of responding to the tumor microenvironment (TME). 1‐methyl‐1*H*‐benzimidazole (BM), an aromatic amine molecule, interacts with β‐CD at pH 7.4 but dissociates at pH 6 or lower due to the protonation of BM (pKa 5.6725).^[^ ^34^ ^]^ On account of the pH difference among normal tissues (pH ≈7.4), TME (pH ≈6.5–7.0), and the intracellular endosome/lysosome (pH ≈4.3–6.8),^[^ ^35^ ^]^ β‐CD (host molecules)‐modified nucleic acids have been reported to link with BM (guest molecules)‐coated SNPs for pH‐responsive drug delivery. For example, Shen et al. modified human epidermal growth factor receptor‐2 (HER2) aptamers with β‐CD and subsequently combined them with BM surface‐functionalized MSNs encapsulating doxorubicin (DOX).^[^ ^36^ ^]^ The FSNPs were able to release DOX under an acidic environment, and the release rate of DOX from the FSNPs reached up to 82.3 ± 5% at pH 4.5, mimicking the environment of cellular lysosomes/endosomes. Furthermore, confocal laser scanning microscope images confirmed that DOX could be delivered into the cytoplasm of SKBR3 cells.

Azobenzene (Azo), as the guest molecule, has been used to modify nucleic acids and then insert them into the host supramolecular β‐CD under normoxic conditions. Interestingly, Azo can be cleaved by hypoxia‐related azoreductase in tumor cells to produce aniline derivatives and then dissociated from hydrophobic inner cavities of β‐CD.^[^ ^37^ ^]^ Tang et al. fabricated a DNA hybridization complex terminated with Azo and then conjugated with β‐CD surface‐modified AuNPs.^[^ ^38^ ^]^ The double strands of the DNA hybridization complex were labeled with Black Hole Quencher‐2 and tetramethylrhodamine (TAMRA), respectively. The fluorescence of TAMRA was clearly quenched by AuNPs, whereas the intensity of that enhanced about 7.1‐fold following incubation with rat liver microsomes/nicotinamide adenine dinucleotide phosphate (simulated hypoxic conditions) for 6 h, which demonstrated Azo‐modified DNA hybridization complex was released from β‐CDs‐coated AuNPs in response to hypoxia.

#### 2.1.2 Electrostatic Interaction

An electrostatic interaction derives from the force of attraction between two oppositely charged molecules. Chitosan is a natural cationic polysaccharide obtained from partially or completely *N*‐deacetylated chitin. With a positive charge at a physiological pH (pH 7.4), chitosan develops electrostatic attraction forces with the negative charge of liposomes and can adhere to the surface of negative liposomes with a high degree of aggregation, as well as neutral liposomes with a much lesser extent aggregation.^[^ ^39^ ^]^ Accordingly, Li et al. conjugated anti‐epidermal growth factor receptor (anti‐EGFR) aptamers with chitosan and demonstrated that the aptamer‐modified chitosan was anchored on the surface of neutral liposomes.^[^ ^40^ ^]^ Similarly, hyaluronic acid, a major anionic glycosaminoglycan component of the extracellular matrix, was reported to provide negative charges for the conjugation of AS1411 aptamers onto the surface of positively charged hybrids composed of CaCO_3_, protamine, and CRISPR‐Cas9 plasmid.^[^ ^41^ ^]^ KALA, a cationic peptide, can combine with negatively charged nucleic acids through electrostatic interaction. Xu et al. used KALA as a bridge to prepare AS1411 aptamer‐decorated protein nanoparticles.^[^ ^42^ ^]^ Specifically, the positively charged KALA/AS1411 complexes were first obtained through incubating AS1411 with excess KALA and then were confirmed to decorate the surface of the negatively charged bovine serum albumin (BSA) nanoparticles. In addition, it has been demonstrated that KALA can facilitate FSNPs transported into the cytoplasm. It occurs probably because cationic KALA drives the outer anionic lipids of the endosome inverted into endocoele to form neutral ion pairs and then promote the formation of the unstable nonmembrane structure to induce endosomal membrane leakage.^[^ ^43^ ^]^

#### 2.1.3 Desthiobiotin‐Avidin Interaction

Avidin can specifically bind with biotin and its analogs (e.g., desthiobiotin). Vitamin H, the endogenous biotin overexpressed in tumor cells,^[^ ^44^ ^]^ can compete for the interaction of desthiobiotin with avidin due to the fact that biotin has a higher binding affinity with avidin than desthiobiotin.^[^ ^45^ ^]^ Accordingly, Li et al. loaded DOX in the pores of MSNs containing Ag_2_S quantum dots (QD@M), along with avidin capping the pores, and further prepared DOX‐loaded QD@M/D‐DNA after the desthiobiotin‐modified survivin antisense oligonucleotide (ASO) (D‐DNA) attachment to the avidin.^[^ ^46^ ^]^ The release rate of DOX from QD@M/D‐DNA without biotin treatment was ≈25%, but that with biotin incubation was increased to 67.44 ± 2.73% under pH 6.5 for 24 h. The authors further found that QD@M/D‐DNA could release DOX in A549 adenocarcinomic cells, and most drug molecules entered the nucleus at 12 h post‐treatment, suggesting that FSNAs prepared based on desthiobiotin–avidin interaction contribute to achieving biotin‐responsive drug delivery.

#### 2.1.4 Other Physical Interactions

As poly adenine (poly‐A) has high coordination capability with AuNPs,^[^ ^47^ ^]^ nucleic acids extended with poly(A) sequences can be attached to AuNPs surface. Moreover, the surface density of nucleic acids on AuNPs can be rationally manipulated by varying poly‐A length. Chen et al. terminated unmethylated cytosine–phosphate–guanosine (CpG) containing oligodeoxynucleotides (ODNs) with different poly‐A length (A5 and A10) to functionalize 15 nm AuNPs. They found the surface density of A5‐CpG ODNs was approximately twofold higher than that of A15‐CpG ODNs on AuNPs under the same conditions.^[^ ^48^ ^]^ In addition, Fang et al. conjugated Sgc8 aptamers with BSA nanoparticles and demonstrated that poly (ϵ‐caprolactone) (PCL)‐*ss*‐cytarabine micelles were surface‐decorated with Sgc8‐BSA via BSA absorption.^[^ ^49^ ^]^

### 2.2 Chemical Conjugation

#### 2.2.1 Bioorthogonal Reactions

Bioorthogonal reactions are two‐component “ligation” reactions that can occur in living systems without interfering with native biochemical processes. To date, three types of bioorthogonal reactions have been reported, including metal‐catalyzed bioorthogonal reactions (e.g., copper‐catalyzed alkyne–azide cycloaddition, ironporphyrin‐catalyzed bioorthogonal reactions), photoclick reactions, and bioorthogonal reactions without catalysts such as Staudinger ligation, strain‐promoted cycloaddition reactions, and inverse electron‐demand Diels–Alder reaction.^[^ ^50^ ^]^ Among these reactions, the Cu(I) catalyzed azide–alkyne cycloaddition (CuAAC) is the most widely applied in the preparation of FSNPs, mainly due to its high selectivity, high yield, high fast rate, and mild reaction conditions.^[^ ^51^ ^]^ CuAAC was initially reported by Sharpless and co‐workers,^[^ ^52a^ ^]^ and Meldal and co‐workers in 2002.^[^ ^52b^ ^]^ For this reaction, the nucleic acid is first modified with an alkynyl group and then conjugated with azide‐functionalized SNPs using Cu(I) catalysis. Accordingly, Chen et al. successfully functionalized azide‐capped MSNs with alkynyl‐modified thrombin aptamers with high efficiency via CuAAC.^[^ ^53^ ^]^ However, the removal of Cu(I) ion can be problematic if nanomaterials contain functional groups able to bind Cu(I) ion, which is potentially toxic to organisms. Thus, strain‐promoted azide–alkyne cycloaddition (SPAAC), referred to as a copper‐free click reaction developed by Bertozzi and co‐workers,^[^ ^54^ ^]^ has become increasingly popular in the coupling of nucleic acids and SNPs. Promoting the reaction of dibenzocyclooctyne (DBCO) containing an alkynyl group with azide was the common strategy of SPAAC. For example, Wang et al. coupled DBCO‐terminated ASO G3139 with azide‐functionalized PEG‐*b*‐PCL amphiphiles and then self‐assembled to form G3139‐functionalized micelles.^[^ ^55^ ^]^ Also, Ferrer et al. directly linked DBCO‐modified nonimmunogenic oligonucleotide (ODN 2138) with azide‐terminated liposomes.^[^ ^31^ ^]^

#### 2.2.2 Amide Bond

The amide bond is generally formed by the reaction of amine and activated carboxylic groups (—COOH). As a result, the nucleic acids terminated with amines or carboxylic groups can be attached to the surface of SNPs premodified with carboxylic or amine groups through amide reaction. Zhao et al. developed the liposomes comprising DSPE‐PEG_2000_‐COOH. The carboxylic groups of liposomes were activated by 1‐ethyl‐3‐(3′‐dimethylaminopropyl) carbodiimide hydrochloride and *N*‐hydroxysuccinimide, and then effectively surface‐decorated by the epithelial cell adhesion molecule (EpCAM) aptamers terminated with amines.^[^ ^56^ ^]^ Similarly, micelles and SPIONs premodified with carboxylic groups or proteins containing many carboxyl groups were demonstrated to be covalently attached by amine‐modified nucleic acids.^[^ ^57^ ^]^

MSNs, whose surface exists silanol, can be easily functionalized by 3‐AminopropylTriethoxySilane (APTES). As the amine source of MSNs, APTES assists MSNs directly coupling with carboxylic‐modified nucleic acids through amide reaction.^[^ ^58^ ^]^ In addition, MSNs decorated with APTES have been reported to link with amine‐modified nucleic acids using polydopamine (PDA) coating,^[^ ^59^ ^]^ succinic anhydride,^[^ ^60^ ^]^ or 4,4′‐azobis (4‐cyanovaleric acid) (AC) as a bridge. Considering that the azide bond of AC, a small thermosensitive molecule, would be broken under 42 °, Sun et al. modified the surface of DOX‐loaded MSNs containing gold nanorods with AC before conjugation with amine‐modified survivin‐targeted DNAzyme.^[^ ^61^ ^]^ When gold nanorods absorbed NIR light and caused light–heat conversion, the generated hyperthermia triggered AC bond breaking, resulting in the release of survivin‐targeted DNAzyme and DOX.

#### 2.2.3 Solid‐Phase Phosphoramidite Chemistry

Solid‐phase phosphoramidite chemistry, invented by Caruthers et al. in 1987, is the commonly used approach for the automatic synthesis of DNA.^[^ ^62^ ^]^ Recently, the solid‐phase phosphoramidite approach has been reported to incorporate hydrophobic ligands at specific positions within nucleic acids. In general, the ligand phosphoramidite is first prepared and then directly coupled onto the 5′‐end of a nucleic acid sequence by the DNA synthesizer. The whole process is carried out on solid support (also called resins), in which controlled pore glass and polystyrene are the most useful. Also, it is automatic, and large excesses of solution‐phase reagents can still be added for the reaction to go to completion and removed easily. As such, this reaction is fast and highly effective. Through this approach, amphiphilic DNA–ligand conjugates are obtained and then self‐assembled to construct DNA micelles. For example, Wu et al. successfully prepared aptamer‐functionalized micelles through the self‐assembly of diacyllipid‐aptamer (specifically binding with adenosine 5′‐triphosphate [ATP]) conjugates.^[^ ^63^ ^]^

#### 2.2.4 Thioether Bond

Thioether bond can be formed through a thiol–maleimide crosslinking reaction that has been used for the linking of nucleic acids to SNPs. The nucleic acids are mostly modified with thiol groups. As thiol groups are easily oxidized to form disulfide bonds that do not react with maleimide groups,^[^ ^64^ ^]^ thiol groups terminated at the end of nucleic acids are first deprotected by reduced agents, such as the commonly used tris(2‐carboxyethyl)phosphine hydrochloride, to produce sulphydryl groups, and then react with maleimide groups coated onto the surface of SNPs, including liposomes,^[^ ^65^ ^]^ micelles,^[^ ^66^ ^]^ and MSNs,^[^ ^67^ ^]^ to form thioether bonds. The reaction proceeds under an oxygen‐free environment to prevent the formation of disulfide bonds before thiol groups conjugation with maleimide groups.

#### 2.2.5 Other Chemical Conjugations

Thiol‐modified nucleic acids can also be absorbed onto the surface of AuNPs and AgNPs through strong metal‐S chemistry.^[^ ^68^ ^]^ Glutathione (GSH) is a small thiol‐based tripeptide composed of glutamic acid, cysteine, and glycine, and Au—S bonds can be cleaved by a high concentration of GSH.^[^ ^69^ ^]^ As GSH is relatively concentrated in the cytoplasm of live cells (1–10 mM) but presents low concentrations in the blood plasma (2–20 μM),^[^ ^70^ ^]^ Zhang et al. functionalized AuNPs with thiol‐modified TLS11a aptamers for GSH‐responsive drug delivery.^[^ ^71^ ^]^ Chlorin e6 (Ce6) was labeled at the 5′end of TLS11a aptamer, and its fluorescence was prequenched by AuNPs. In this nanoplatform, the fluorescence intensity of Ce6 was tested in the presence of 10 mM DL‐Dithiothreitol (mimicking intracellular GSH) at different times. The results showed that Au—S enabled significantly enhanced releasing efficacy of drugs (8.5‐fold higher) at the 5 h post‐incubation under high GSH concentration. Notably, the formed Au—S bond would be very stable in physiological conditions, for the results that the fluorescence intensity of Ce6 was scarcely influenced in the absence of DL‐Dithiothreitol over time.

## 3 FSNPs for Cancer Therapy

Due to the programmability of the nucleic acid sequence, a series of FNAs have recently been discovered and synthesized. Except for features of nucleic acids, such as excellent intrinsic biocompatibility, biodegradability, and negative charge under physiological conditions, FNAs are capable of binding with biological targets or exhibit stimuli‐responsive structures transformation, catalysis, or therapeutic capabilities. Among FNAs, aptamer, i‐motif, DNAzyme, ASO, and CpG ODN are widely used in the surface modification of spherical nanomaterials for enhanced antitumor effect and lower systemic toxicity. Here, we elaborate on the characteristics of these FNAs and the applications of relevant FSNPs in cancer therapy.

### 3.1 Aptamer‐Decorated SNPs

Aptamers are synthetic, single‐stranded DNA/RNA oligonucleotides that can specifically recognize their target molecules through folding unique secondary or 3D structures. Mostly, aptamers are obtained via combinational chemistry of systemic evaluation of ligand by exponential enrichment (SELEX).^[^ ^72^ ^]^ The RNA aptamer selection was originally carried out by Ellington and Tuerk,^[^ ^12^, ^73^ ^]^ but DNA aptamer has been recently rapidly developed for several advantages of chemical stability, cost‐effectiveness, and obviation of a reverse‐transcription step during the SELEX process.^[^ ^74^ ^]^

Aptamers as FNAs can selectively bind target molecules of interest, such as metal ions,^[^ ^75^ ^]^ small organic molecules,^[^ ^76^ ^]^ proteins,^[^ ^77^ ^]^ and even whole cells.^[^ ^78^ ^]^ On account of higher binding affinity in target cells than nontarget cells,^[^ ^79^ ^]^ aptamers have been used as targeting ligands of SNPs for delivering drugs to the tumor site. In addition, aptamer, capable of binding with specific intracellular molecules in the tumor cells,^[^ ^80^ ^]^ can function as a responsive ligand to modify SNPs for controlled drug release.

#### 3.1.1 Targeted Drug Delivery

To date, numerous tumor‐associated aptamers have been reported to modify SNPs for targeted drug delivery (**Table** **2**). These aptamers are mainly divided into four types according to their targets: 1) targeting overexpressed proteins on cancer cells or cancer cells; 2) targeting mitochondria; 3) targeting angiogenesis; 4) simultaneously targeting cancer cells and other cancer‐associated cells.

**Table 2:** Aptamer‐decorated SNPs for targeted drug delivery

Types of target		Aptamer	Target	Conjugated spherical nanoparticles	Cargo	Tumor cells tested	Ref.
Cancer cells	Overexpressed proteins	AS1411	Nucleolin	AuNPs	AS1411	HeLa	[84]
				AuNPs	TMPyP_4_, DOX	MCF‐7R	[86]
				Micelles	hemin, tetraoxane	HepG2	[89]
		MAGE‐A3	MAGEA‐3111‐125	MSNs	Afatinib	CL1‐5	[93]
		Gint4.T	U87MG	Micelles	Nile red	U87MG	[96]
		Apt1	CD44	Liposomes	Luciferase siRNA	MDA‐MB‐231	[27]
		EpCAM	EpCAM	Liposomes	MiR‐139‐5p	HCT8	[56]
		HB5	HER2	Albumin	Curcumin	SK‐BR3	[57c]
		MUC1	MUC1	MSNs	DOX	MCF‐7	[60]
		Sgc8	PTK7	Fe_3_O_4_	DOX	A549	[97a]
				Micelle	DOX	CCRF‐CEM cells	[97b]
Cancer cells	Overexpressed proteins	GBI‐10	Tenascin‐C	Liposomes	Gadolinium	C6 glioma	[98]
		EGFR	EGFR	Liposomes	Bcl‐2 and PKC‐ι siRNA	MDA‐MB‐231	[99]
		SZTl01	PSMA	Liposomes	TPEN	PCa	[100]
	Cancer cells	TLS11a	HepG2	AuNPs	Ce6	HepG2	[71]
		SRZ1	4T1	Liposomes	DOX	4T1	[101]
		Apt S1	MDA‐MB‐231	Micelles	Photosensitizer R16FP	MDA‐MB‐231	[102]
		As42	A549	AuNPs	AuNPs	A549	[103a]
		S13	A549	Micelles	ferrocene	A549	[103b]
Mitochondria		Cyt c	mitochondrion	Micelles	DOX	HeLa	[108]
Angiogenesis		ENG	ENG	Liposomes	mIP‐10 plasmid	mTECs	[111]
Cancer cells and cancer‐associated cells		T1	TME	Liposomes	DOX	MDA‐MB‐231 PMN‐MDSCs	[113]

##### Aptamers Targeting Overexpressed Proteins on Cancer Cells or Cancer Cells

Nucleolin, a nonribosomal nucleolar protein, is overexpressed on the surface of various cancer cells, such as lung cancer, breast carcinoma, and glial carcinoma. The elevated expression of nucleolin facilitates the carcinogenesis, proliferation, survival, infiltration, and metastasis of cancer cells.^[^ ^81^ ^]^ As such, nucleolin has been used as a potential anticancer therapeutic target. AS1411, known as a classical non‐SELEX aptamer that was occasionally discovered and designed by Bates et al. in the late 1990s, can specifically interact with nucleolin.^[^ ^82^ ^]^ Consequently, it has been demonstrated that AS1411 aptamers can target a wide variety of cancer cells with overexpressed nucleolin and inhibit their proliferation. Importantly, after conjugation with SNPs, the antiproliferation efficacy to cancer cells of AS1411 is enhanced,^[^ ^83^ ^]^ which might contribute to the fact that the densely distributed AS1411 on the surface of SNPs are easier to be internalized by tumor cells than free AS1411. Kabirian–Dehkordi et al. constructed AS1411‐functionalized AuNPs (AS1411‐AuNPs) and found AS1411‐AuNPs could inhibit the expression of nucleolin and the proliferation of HeLa cells.^[^ ^84^ ^]^ Although the AS1411‐AuNPs‐induced disorganization of the nucleolar structure was also observed in cells treated with nucleolin siRNA, the alteration of genes implicated in the cell cycle and the cell‐cycle blockage was different from that in nucleolin‐knocked down cells, indicating the antiproliferation mechanism of AS1411‐AuNPs seems independent of nucleolin.

In addition, with sequence, 5′‐GGTGGTGGTGGTTGTGGTGGTGGTGG‐3′, AS1411 aptamer binding to its complementary DNA (cDNA) can form double‐stranded GC base pairs for DOX loading.^[^ ^85^ ^]^ Particularly in the presence of K^+^, the AS1411 aptamer can form a G‐quadruplex structure and interact with several photosensitizers such as tetrakismethylpiridiniumylporphine (TMPyP_4_),^[^ ^86^ ^]^ zinc phthalocyanine (ZnPc),^[^ ^87^ ^]^ and Ce6 or hemin,^[^ ^88^ ^]^ which contain a large π‐planar structure for binding to G‐quadruplexes via strong π–π stacking interactions. As such, Shiao et al. developed AS1411‐functionalized AuNPs for the codelivery of two different anticancer drugs.^[^ ^86^ ^]^ The AS1411 aptamer was extended with a T_6_ (CGATCGA)_3_ sequence, of which the 6 extra T bases ensure the formation of G‐quadruplex structure to load TMPyP_4_, followed by hybridization with T_10_ (TCGATCG)_3_ sequence to form GC base pairs for DOX binding. The thiol‐terminated T_10_ (TCGATCG)_3_ sequence not only facilitates AS1411 extended with double‐stranded DNA ds (AS1411) approached to the AuNPs but also keeps the G‐quadruplex structure of ds (AS1411) extend away from the AuNPs surface. The ds (AS1411)–AuNPs conjugates achieved high loading efficiency of TMPyP_4_ (87 ± 4%) and DOX (81 ± 7%) and codelivered them to DOX‐resistant MCF‐7R breast cancer cells for synergetic therapy.

**Figure 1:**
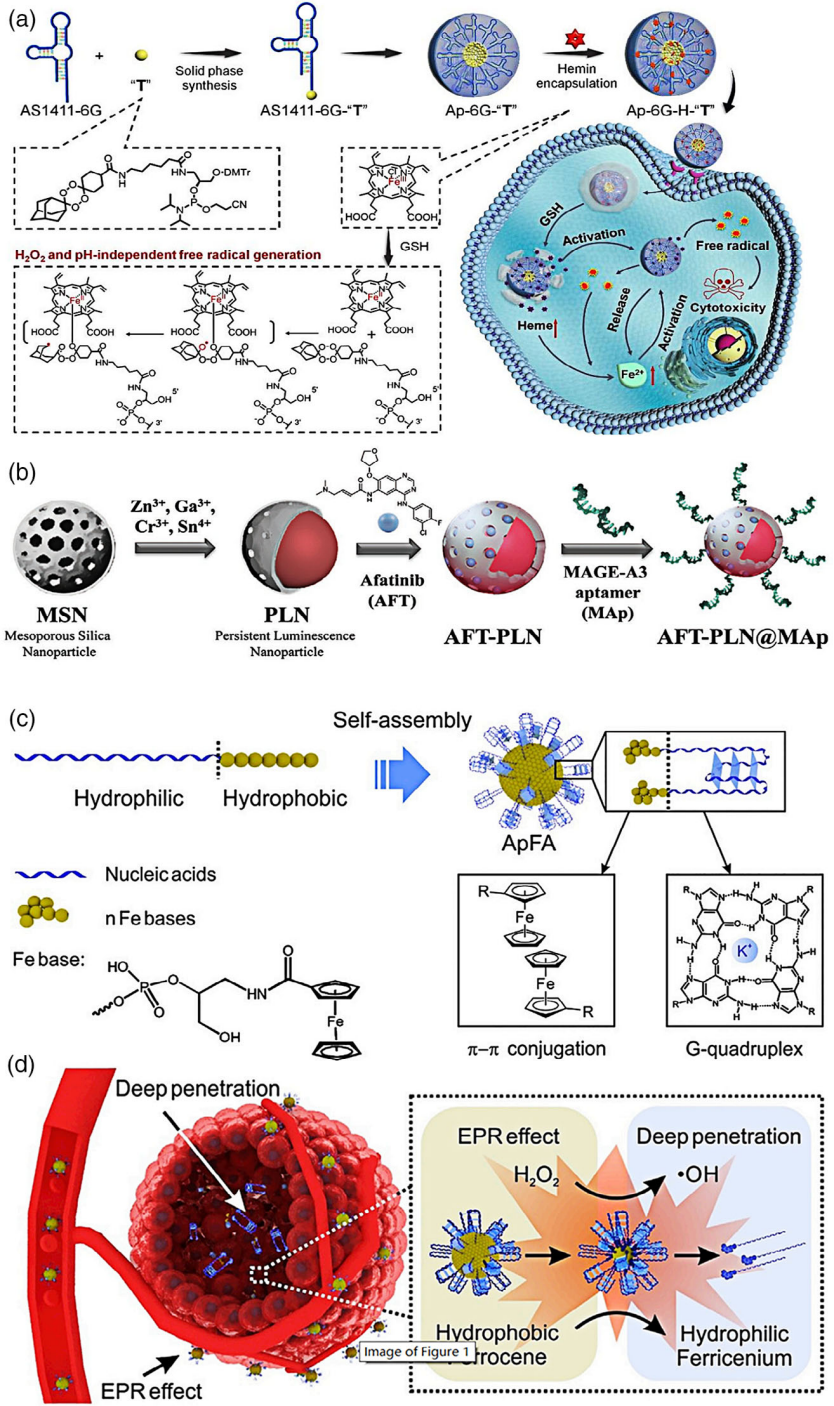
a) The AS1411‐prodrug conjugate self‐assembled micelles encapsulating hemin for the depletion of cancerous antioxidant defense and enhanced CDT. Reproduced with permission.^[^ ^89^ ^]^ Copyright 2019, American Chemical Society. b) Schematic illustration for the fabrication process of AFT‐PLN@Map. Reproduced under the terms of the CC‐BY 4.0 license.^[^ ^93^ ^]^ Copyright 2020, The Authors, published by Wiley‐VCH. c) Schematic illustration for the construction of S13 aptamer‐modified micelles containing numbers of ferrocene (ApFA). d) The size‐changeable ApFA micelles for tumor accumulation and deep penetrating cancer therapy in response to the TME. c,d) Reproduced with permission.^[103b]^ Copyright 2019, Elsevier Inc.

Hemin, an iron‐containing porphyrin, can be reduced into heme (Fe^2+^) by intracellular GSH, as a major antioxidant defense of cancer cells, which limits the therapeutic efficiency of oxidation therapy, including photodynamic therapy (PDT) and chemodynamic therapy (CDT). Xuan et al. developed the AS1411‐prodrug conjugate self‐assembled micelles encapsulating hemin to deplete cancerous antioxidant defense and enhance CDT (**Figure** **1**a).^[^ ^89^ ^]^ The prodrug, Fe^2+^‐activable tetraoxane (“T”), was conjugated with AS1411 using six G bases as the linker, followed by self‐assembly to form Ap‐6G‐“T” micelles. Finally, hemin was loaded in both the G‐quadruplexes structure of AS1411 shell and hydrophobic “T” core of the ApPdC micelles. Using ApPdC micelles with a random DNA sequence as a negative control, they found that ApPdC micelles with one or two “T” motifs were preferable for decreasing nonspecific membrane insertion of DNA micelles. Once entering HepG2 cells, intracellular GSH reduced the loaded hemin (Fe^3+^) into heme (Fe^2+^), which led to the decreased intracellular GSH content, and then activated “T” to self‐cycling generate toxic C‐based free radicals in the independence of H_2_O_2_ and pH. Experimental results demonstrated that Ap‐6G‐H‐“T” exhibited high serum and nuclease stability, as well as achieved enhanced therapeutic effect of CDT compared with hemin‐free Ap‐6G‐“T”.

Melanoma‐associated antigen‐A3 (MAGE‐A3) is a highly expressed cancer/testis antigen in tumors. It was initially discovered in melanoma and also expressed in various other cancers, such as esophageal carcinomas, nonsmall cell lung cancer, gastric carcinomas, multiple myeloma, and bladder cancer.^[^ ^90^ ^]^ Moreover, the expression of MAGE‐A3 has often been associated with malignancy and metastasis of cancer.^[^ ^91^ ^]^ Therefore, MAGE‐A3 is considered as an attractive target for metastatic cancer therapy.^[^ ^92^ ^]^ For example, Chan et al. modified MSNs with MAGE‐A3 aptamer (Figure 1b).^[^ ^93^ ^]^ The malignant and metastatic lung adenocarcinomic cell CL1‐5 exhibited a higher uptake efficiency of MAGE‐A3‐functionalized MSNs than normal lung cell Beas2B. Significantly, the MSNs were doped with several metal ions, including zinc (Zn), gallium, chromium, and stannum, to generate near infrared‐persistent luminescence nanomaterials (ZGOCS@MSNs), which emitted near‐infrared (NIR) light for several hours under irradiation with UV light. The authors found that MAGE‐A3‐modified ZGOCS@MSNs was capable of long‐term tracking lung cancer metastasis, as well as delivering afatinib, the first‐line used antitumor drug, which was loaded into the pore of ZGOCS@MSNs, to in situ tumor and metastatic tumor, causing the inhibition of CL1‐5 tumor cells growth and metastasis.

Glioma is the most common malignant tumor of the central nervous system. A great challenge in glioma therapy lies in the lack of successful vehicles capable of delivering drugs to brain tumor sites in the presence of BBB and blood–brain tumor barrier (BBTB), as the major obstacle for the delivery of drugs into brains, which greatly limit therapeutic efficacy of glioma treatment.^[^ ^94^ ^]^ Gint4.T aptamer can target glioblastoma cells U87MG through specifically recognizing overexpressed platelet‐derived growth factor receptor β, and SNPs surface‐functionalized with dense and highly oriented nucleic acids (namely SNAs) was demonstrated to transcytose across different biological barriers, including BBB and BBTB.[^16^, ^95^] Thus, Xiao et al. constructed U87MG‐specific aptamer‐functionalized micelles through self‐assembly of polystyrene‐*b*‐DNA copolymers, which are highly expected to efficiently cross BBB for brain tumor therapy.^[^ ^96^ ^]^ They proved that micelle‐based SNAs could cross the BBB through scavenger receptor‐mediated transcytosis, shown by in vitro competition inhibition assay. Furthermore, in contrast with PEG as the shell of micelles, they found that U87MG‐specific aptamer‐functionalized micelles showed a 4.5‐fold higher traversing efficiency and further showed a 4.1‐fold more internalization to U87MG cells than those treated with non‐U87MG‐specific single‐stranded DNA conjugated as a negative control, which will facilitate the further therapy of brain tumors.

There are many other aptamers against other overexpressed receptor proteins on the cancer cell surface, including a cluster of differentiation‐44 (CD44),^[^ ^27^ ^]^ EpCAM,^[^ ^56^ ^]^ HER2,^[57c]^ mucin‐1(MUC1),^[^ ^60^ ^]^ proteintyrosinekinase‐7 (PTK7),^[^ ^97^ ^]^ tenascin‐C,^[^ ^98^ ^]^ EGFR,^[^ ^99^ ^]^ prostate‐specific membrane antigen (PMSA).^[^ ^100^ ^]^ These receptors exist in most cancer cells, and the corresponding aptamer‐modified SNPs have been applied in the targeted treatment of different types of cancer. To confirm that aptamers are able to interact with cell‐membrane receptors after decoration onto the surface of SNPs, there have been the following two ways to be reported. The first one is to use normal cells or cancer cells without or with no significant targeted receptor proteins as a negative control, and positive‐selection cells or targeted receptor proteins overexpressed tumor cells as a positive control. The second one is to pretreat positive cells with targeted receptor competitive inhibitors. For example, Kim et al. fabricated anti‐EGFR aptamer‐functionalized liposomes.^[^ ^99^ ^]^ Using EGFR‐negative MDA‐MB‐453 as a negative control and EGFR‐expressing MDA‐MB‐231 breast cancer cells as a positive control, results demonstrated highly efficient cell internalization in MDA‐MB‐231 rather than MDA‐MB‐453. Furthermore, anti‐EGFR aptamer‐functionalized liposomes displayed significantly diminished binding to pretreated cells with EGFR competitive inhibitors, cetuximab. These results indicate that the second structure of anti‐EGFR aptamer attached on the surface of liposomes is fold correctly and enable cell internalization via the EGFR‐mediated pathway. However, the second way is not suitable for these aptamers, such as TLS11a,^[^ ^71^ ^]^ SRZ1,^[^ ^101^ ^]^ AptS1,^[^ ^102^ ^]^ and As42 or S13,^[^ ^103^ ^]^ of which targeted receptor proteins on the positive‐selection cells are not clear. Accordingly, SNPs conjugated with these aptamers just enable the delivery of drugs to specialized targeted cancer cells.

The major challenge in solid tumor therapy is the malformation of blood vessels and dense extracellular matrix in the TME, as the barriers of large‐sized nanoparticles (≈100 nm) interstitial delivery for deep tumor penetration.^[^ ^104^ ^]^ Recently, size‐switchable therapeutic nanoparticles capable of responding to TME conditions have been developed to improve therapeutic outcomes in solid tumors.^[^ ^105^ ^]^ Tan et al. constructed S13 aptamer‐functionalized micelles containing numbers of ferrocene (ApFA) for deep penetrating cancer therapy in response to acidic pH and high‐H_2_O_2_ dual‐TME.^[103b]^ S13 aptamer, a kind of guanine‐rich oligonucleotides targeting A549 cells, can form a G‐quadruplex structure in the presence of K^+^. Seven numbers of ferrocene, a hydrophobic molecule composed of two parallel cyclopentadiene rings, was conjugated with nucleic‐acid sequence and then self‐assembled to form ApFA micelles with ≈100 nm, of which entire structure could be stabilized by G‐quadruplexes and π–π conjugated ferrocene moieties (Figure 1c). It was demonstrated that the ApFA micelles accumulated at tumor sites. Moreover, after treatment with H_2_O_2_ at pH 6.0 mimicking TME, hydrophobic ferrocene could be oxidized into hydrophilic Fe (Cp)^2+^ via a Fenton‐like reaction, which decreased the structural stability of the micelles, resulting in the size of S13 aptamer‐modified micelles shrinking from about 100–10 nm. Also their enhanced penetration behaviors were observed in the tumor tissue derived from A549‐tumor‐bearing BALB/c nude mice (Figure 1d). Furthermore, ApFA micelles loaded with glucose oxidase, which can catalyze the glucose in the tumor region into massive H_2_O_2_ to promote the high production of cytotoxic hydroxyl radicals from ferrocene, achieved significant antitumor therapeutic efficiency.

##### Aptamer Targeting Mitochondria

Mitochondria, known as the energy powerhouse of a cell, decisively regulate cancer development and progression in several aspects, including metabolic reprogramming, acquisition of tissue infiltration and metastatic capability, and apoptosis resistance.^[^ ^106^ ^]^ Mitochondria in chemotherapeutic drug‐resistant cancer cells can exhibit high membrane potentials and abnormal cellular metabolism, which are associated with tumor cell apoptosis resistance and cause an insufficient therapeutic effect.^[^ ^107^ ^]^ Cytochrome c (Cyt c), a small hemoprotein, is normally bound to the inner mitochondrial membrane. The discovery of the aptamer targeting Cyt c facilitates the intracellular delivery of drugs toward mitochondria. Accordingly, Chen et al. demonstrated the Cyt c aptamer‐functionalized DOX‐loaded dendrigraft poly‐l‐lysines nanoparticles could dramatically decrease the mitochondrial membrane potential and induce prominent apoptosis of DOX‐resistant HeLa/ADR cells, whereas no significant killing effect was observed in free DOX‐treated cells, indicating that delivering drugs toward mitochondria via Cyt c aptamer can selectively kill drug‐resistant cells.^[^ ^108^ ^]^

##### Aptamer Targeting Angiogenesis

Angiogenesis, namely, the formation of new blood from preexisting vasculature, plays essential roles in tumor growth and hematogenous metastasis.^[^ ^109^ ^]^ Endoglin (ENG), a crucial angiogenic marker that can stimulate angiogenesis, is rarely expressed on normal blood vessels.^[^ ^110^ ^]^ Thus, Yang et al. conjugated aptamer capable of targeting ENG (ENG‐Apt) onto the surface of liposomes encapsulating the expression plasmid of the mouse interferon (IFN)‐induced protein‐10 (mIP‐10) gene (mIP‐10‐LPs),^[^ ^111^ ^]^ which can not only efficiently chemoattract cytotoxic T cells (e.g., CD8^+^ T cells in particular) toward local tumor tissues but also inhibit tumor growth and angiogenesis.^[^ ^112^ ^]^ Compared with mIP‐10‐LPs, the uptake of ENG‐Apt functionalized mIP‐10‐LPs (ENG‐Apt/ mIP‐10‐LPs) in mouse tumor vascular endothelial cells (mTECs) enhanced ≈16 times, demonstrating high tumor neovasculature specificity of ENG‐Apt. In addition, flow cytometry assay confirmed that ENG‐Apt/mIP‐10‐LPs recruited both endogenous and exogenous CD8^+^ T cells around melanoma tumor vasculatures, resulting in a prolonged survival time in melanoma tumor‐bearing mice. These results indicate that SNPs functionalized with angiogenesis‐targeted aptamers serve as a promising strategy for cancer therapy.

##### Aptamer Simultaneously Targeting Cancer Cells and Cancer‐Associated Cells

In general, an aptamer just targets one cancer cell or cancer‐associated cell. Recently, Liu and co‐workers identified a DNA aptamer (T1) that could simultaneously target breast cancer cells and myeloid‐derived suppressor cells (MDSCs).^[^ ^113^ ^]^ The latter one, a heterogeneous population of immature myeloid cells that expand during cancer, can promote tumor growth and metastasis through suppressing T cell responses,^[^ ^114^ ^]^ and even hinder the anti‐cancer activity of immune checkpoint inhibitors.^[^ ^115^ ^]^ Notably, DOX efficiently eliminated and inactivated MDSCs, leading to a transformation of the TME toward an immunostimulatory state.^[^ ^116^ ^]^ Therefore, Liu et al. constructed a T1‐modified liposomal DOX (T1‐DOX) for breast cancer therapy.^[^ ^113^ ^]^ Compared to liposomal DOX conjugated to a random scrambled aptamer sequence as a negative control, T1‐DOX displayed an increased accumulation to 4T1 breast cancer tumor, the dramatically decreased intratumoral population of MDSCs to facilitate a more immunoactive TME, and thus enhanced efficacy in the inhibition of 4T1 breast cancer growth. These results are attributed to the dual targeting ability of T1 aptamer to breast cancer cells and MDSCs.

#### 3.1.2 Controlling Drug Release

ATP is well‐known as an intracellular trigger, probably because of the greater ATP levels within tumor cells (1–10 mM) than those in the extracellular environment (<5 μM),^[^ ^117^ ^]^ as well as normal cells resulted from the increased glycolysis.^[^ ^118^ ^]^ Therefore, Shen et al. designed ATP aptamer‐functionalized nanophotosensitizers (Apt‐HyNP/BHQ_2_) for ATP‐activated PDT.^[^ ^119^ ^]^ First, TMPyP‐Zn‐QD, rhodamine 6G (R6G), DSPE‐PEG_2000_‐OMe, and DSPE‐PEG_2000_‐NH_2_ were mixed with each other to afford HyNPs. Then, AS1411 and a complementary cDNA of ATP aptamer were attached on the surface of the HyNPs, followed by hybridization of a black hole quencher2‐labeled ATP aptamer to obtain Apt‐HyNP/BHQ_2_. The fluorescence of both two kinds of photosensitizers (TMPyP, R6G) was quenched by BHQ_2_. Also the formed nanophotosensitizers Apt‐HyNP/BHQ_2_ exhibited low PDT capacity but could be activated by the high concentration of intracellular ATP after nucleolin receptor‐mediated uptake by HeLa cells. In vivo results demonstrated that Apt‐HyNP/BHQ_2_ exhibited real‐time monitoring of intracellular ATPs and excellent tumor eradication capability. This work provides a promising strategy for the design of precise theranostic nanoplatforms in cancer therapy. In addition, ATP aptamer, with sequence, 5′‐ACCTGGGGGAGTATTGCGGAGGAAGGT‐3′, can form DNA duplex rich in GC base pair for DOX binding, as well as releasing in response to a high ATP level inside tumor cells.^[^ ^120^ ^]^

### 3.2 I‐Motif‐Decorated SNPs

I‐motif is a quadruple structure with two antiparallel duplexes that were discovered in 1993.^[^ ^13^ ^]^ At neutral pH, the i‐motif‐forming sequence, a kind of cytosine‐rich oligonucleotides, hybridizes with its complementary sequence to form G–C duplexes, in which DOX could be rapidly and efficiently loaded.^[^ ^121^ ^]^ But under an acid environment, the i‐motif‐forming sequence is capable of folding into an i‐motif structure, resulting in the release of DOX. The transformation of the i‐motif‐forming sequence from a single strand to a tetraplex structure (C‐quadruplex) was attributed to the fact that protonated cytosine under acidic conditions preferentially binds other cytosine bases rather than guanine bases, and the formed cytosine‐protonated cytosine base pairs further intercalate with each other.^[^ ^13^ ^]^ Interestingly, when increasing to neutral pH, the tetraplex structure returns to a single‐strand conformation caused by deprotonation of cytosines.^[^ ^122^ ^]^ Therefore, i‐motif‐forming sequences are well‐suited to construct pH‐responsive nanoplatforms for the controlled release of drugs, such as i‐motif‐equipped AuNPs.^[^ ^123^ ^]^

**Figure 2:**
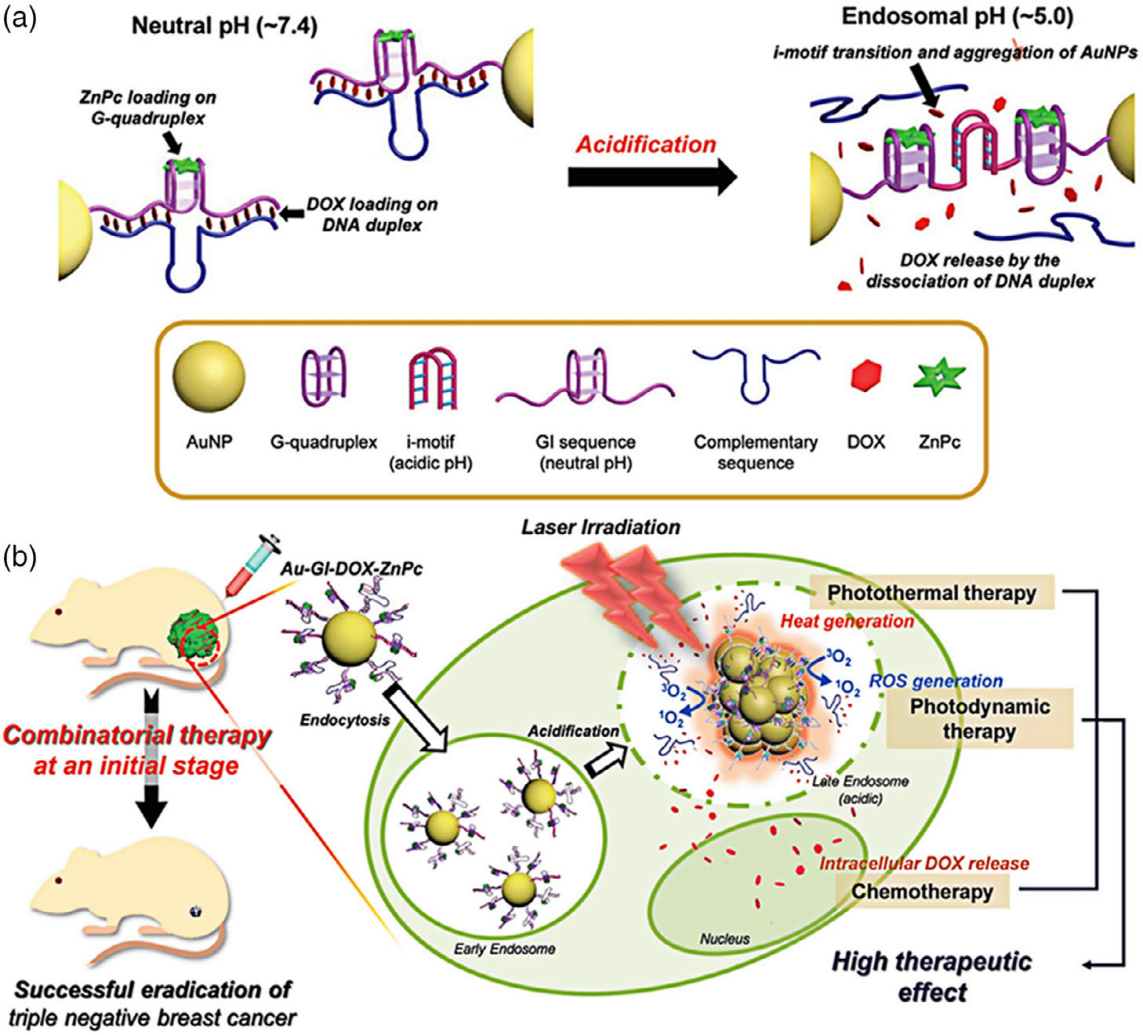
I‐motif‐based aggregatable AuNPs (Au‐GI). a) The design of Au‐GI nanoparticles and mechanism of AuNPs aggregation based on acidic pH. b) Combinatorial photothermal, photodynamic, and chemotherapy of Au‐GI for the successful eradication of triple‐negative breast cancer. a,b) Reproduced with permission.^[^ ^125^ ^]^ Copyright 2017, Wiley‐VCH.

Apart from controlling drug release, the pH‐dependent reversible switch of the i‐motif‐forming sequence from a single strand to the tetraplex structure has been used to induce the aggregation of small‐sized AuNPs (less than 20 nm), which exhibits lower NIR absorption and poorer photothermal conversion efficiency than aggregated AuNPs.^[^ ^124^ ^]^ For example, Park et al. designed (i‐motif)‐based aggregatable AuNPs.^[^ ^125^ ^]^ First, functional DNA containing G‐quadruplex and partial i‐motif‐forming sequence, which is located at the two ends of the G‐quadruplex structure, was designed and synthesized. Then, functional DNA (GI) was combined with its cDNA and was chemically conjugated onto the surface of AuNPs (Au‐GIs). Finally, a photosensitizer (ZnPc) and DOX were loaded into the G‐quadruplex and G–C duplex, which was formed by the hybridization of the i‐motif and its cDNA, respectively (**Figure** **2**a). When the GI‐decorated AuNPs (Au‐GI) were internalized by cancer cells, the partial i‐motif‐forming sequences derived from nearby single Au‐GIs could combine with each other and form i‐motif structures through cytosine^+^–cytosine base pairing at acidic endosomal pH, resulting in the aggregation of Au‐GIs and the release of DOX. Notably, the aggregated Au‐GIs with a size of more than 500 nm efficiently generated heat under NIR irradiation and exhibited photothermal tumor ablation. Ultimately, the triple combinatorial antitumor therapy of DOX‐induced chemo, ZnPc‐induced photodynamic, and aggregated AuNP‐induced photothermal therapy was achieved (Figure 2b).

Although aggregated AuNPs exhibited excellent photothermal conversion efficiency, the poor biodegradable and unfitted for renal clearance, resulted from their larger sizes (more than 20 nm), might cause side effects on normal cells.^[^ ^126^ ^]^ Considering the excellent photothermal conversion efficiency of aggregated AuNPs and the moderate biocompatibility of small‐sized AuNPs, Zhang et al. designed a smart nanoagent system in which the conversion between aggregation and dissociation of AuNPs carries on according to different pH.^[^ ^127^ ^]^ Each AuNP (18 nm) was modified with a ternary DNA complex composed of rC‐DNA, rG‐DNA (r = rich), and ATP aptamer, of which CG duplex formed via Watson–Crick hybridization allows for DOX molecules loading. The FNA‐modified DOX‐loaded AuNPs could be delivered into HeLa cells and rapidly release DOX under the dual stimuli of high ATP and acidic pH. Simultaneously, the intermolecular i‐motif structure was formed and caused intracellular AuNP aggregates (195 nm), which displayed high photothermal conversion efficiency confirmed by both in vitro and in vivo results. More importantly, the formed AuNP aggregates would reversibly disassociate to small‐sized AuNPs under a neutral extracellular environment so as to promote post‐treatment renal clearance of FNA‐modified AuNPs. In conclusion, i‐motif formation in response to endosomal pH can induce AuNPs aggregation to enhance the photothermal effect, as well as unfolding in response to neutral extracellular environments can trigger disassociation of AuNPs aggregation to reduce adverse effects.

### 3.3 DNAzyme‐Decorated SNPs

DNAzyme is a synthetic single‐stranded catalytic DNA molecule that was first presented by Breaker and Joyce in 1994.^[^ ^128^ ^]^ On account of their excellent catalytic activities and well‐characterized structures, RNA‐cleaving DNAzyme, capable of catalyzing the cleavage of targeted RNA substrates, are widely used. Typically, RNA‐cleaving DNAzymes compose of three segments, including a catalytic domain for the binding of metal ions as cofactors such as manganese (Mn^2+^), Zn^2+^, magnesium (Mg^2+^), an active site, and two flanking substrate‐recognition domains that enable complementation to RNA substrate through Watson–Crick base pairing.^[^ ^129^ ^]^ By changing the sequence of the substrate‐recognition domains, DNAzyme can specifically bind the desired RNA substrate and efficiently catalyze its hydrolysis.

**Figure 3:**
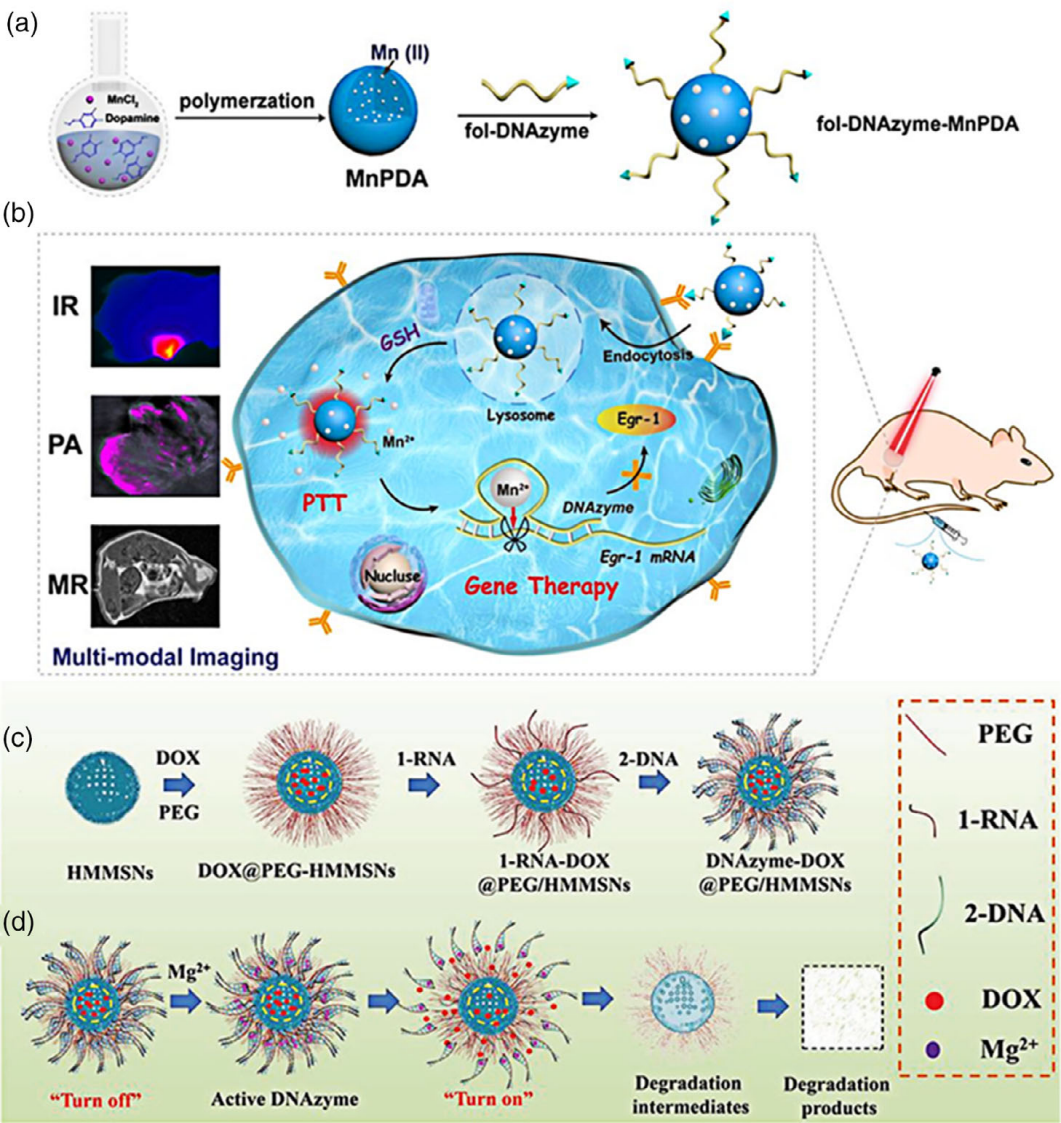
a) Schematic illustration for the preparation of fol‐DNAzyme‐MnPDA nanoparticles. b) The fol‐DNAzyme‐MnPDA nanoparticles for gene‐photothermal synergistic therapy and multimode imaging. a,b) Reproduced with permission.^[^ ^133^ ^]^ Copyright 2018, American Chemical Society. c) Schematic illustration for the Mg^2+^‐dependent DNAzyme‐decorated HMMSNs synthetic process. d) The DNAzyme‐DOX@PEG/HMMSNs for responsive drug release and biodegradation. c,d) Reproduced with permission.^[^ ^138^ ^]^ Copyright 2018, Wiley‐VCH.

DNAzymes are generally obtained by in vitro selection.^[^ ^14^, ^130^ ^]^ The “10‐23” DNAzymes, derived from the 23rd clone from the 10th round of in vitro selective amplification, can cleave almost cytosolic‐targeted RNA substrates containing purine–pyrimidine junctions and have been extensively applied to remove cancer‐causing gene product.^[^ ^131^ ^]^ ED5, one type of “10‐23” DNAzymes capable of targeting and cleaving early growth response‐1 (Egr‐1) mRNA, can block proliferation, migration, and chemo‐invasion of human breast carcinoma cells, potentially serving as an antitumor drug.^[^ ^132^ ^]^ Nevertheless, multifunctional vehicles for highly efficient delivery and sufficient release of ED5 are deficient. Feng et al. used spherical polydopamine‐ Mn^2+^ (MnPDA) nanoparticles as delivering vehicles of ED5, which was directly conjugated on the surface of MnPDA, as well as terminated with folate (fol) for tumor targeting (**Figure** **3**a).^[^ ^133^ ^]^ Apart from protecting ED5 from intracellular degradation, the MnPDA nanoparticles were capable of GSH‐responsive self‐supplying Mn^2+^, as the cofactor to DE5 to catalyze the reaction more efficiently. Results demonstrated that fol‐DNAzyme‐MnPDA conjugates could more effectively cleave Egr‐1 mRNA, of which level was reduced by 80%, than free fol‐ED5. Significantly, the MnPDA vehicles enabled multimodal imaging, composed of magnetic resonance imaging, photoacoustic, and NIR thermal imaging, and achieved excellent photothermal therapy in combination with gene therapy of ED5 DNAzyme (Figure 3b).

In addition, Rouge and co‐workers developed a DNA–surfactant nanocapsule for GATA‐3 targeting DNAzyme (hgd40) intracellular delivery and gene regulation.^[^ ^134^ ^]^ The surfactant as a hydrocarbon tail first self‐assembled, and its surface neighboring alkynes were crosslinked using a diazido ester crosslinker. The alkyne‐terminated surfactant was then surface‐functionalized with the thiolated DNA, which acts as an anchor for DNAzyme attachment using T4 DNA ligase to form a DNAzyme–surfactant nanocapsule. The GATA‐3 is a proinflammatory transcription factor that is highly expressed in breast carcinomas. Thus, MCF‐7 cells were used as a model to evaluate the cellular uptake and gene‐regulation capability of the GATA‐3‐targeted DNAzyme–surfactant nanocapsule. Results showed that the DNAzyme hgd40‐surfactant nanocapsule was effectively internalized by MCF‐7 cells without the use of transfection agents. Because the diazido ester crosslinker can be cleaved by endosomal esterases, the nanocapsule disassembly could be achieved within the cell's endosome and thus release DNz–surfactant molecules to traverse lipid bilayers on the outside of endosomes, leading to escape endosomes. Consequently, the DNAzyme hgd40‐surfactant nanocapsule resulted in persistent knockdown of the GATA‐3 mRNA.

PDT is a promising and noninvasive therapeutic modality for cancer. It can produce cytotoxic reactive oxygen to induce cancer cell necrosis and/or apoptosis by consuming oxygen under photosensitizers and laser irradiation. Notably, PDT can upregulate the expression of survivin, which is associated with cancer cell survival and resistance, resulting in the limited therapeutic effect of PDT.^[^ ^135^ ^]^ Thus, Jin et al. developed an upconversion nanoplatform loaded with photosensitizers TMPyP_4_ and survivin‐targeted DNAzyme for enhanced PDT.^[^ ^136^ ^]^ The nanoplatfrom was first constructed through electrostatic absorption of a long single‐stranded DNA (ApDz), comprising tens to hundreds of tandem repeats of AS1411 aptamer and DNAzyme, onto the surface of upconversion nanoparticles (NaYF_4_:Yb, Er), as photosensitizers energy donors to trigger PDT under NIR light, followed by TMPyP_4_ binding to the G‐quadruplex of AS1411 aptamers. Consequently, the formed upconversion nanoplatform significantly inhibited the expression of survivin and exhibited excellent antitumor response, suggesting that combined with DNAzyme‐based gene therapy is a promising strategy for greatly enhanced PDT.

In addition, metal ion‐dependent DNAzyme‐functionalized SNPs have been appealing in stimuli‐responsive drug delivery. For example, He et al. reported Zn^2+^‐dependent DNAzyme‐modified AuNPs with acid‐decomposable ZnO QDs as Zn^2+^ providers.^[^ ^137^ ^]^ The AuNPs were first surface‐decorated with AS1411 aptamer and Zn^2+^‐dependent DNAzyme (E), followed by the hybridization between the DNAzyme and its substrate (S) strand extended with antisense DNA anti‐miR‐21. The formed double‐stranded GC pairs and negatively charged DNA chains allowed for DOX and positively charged ZnO QDs loading, respectively. Results demonstrated that the GNPs‐ES@QDs could produce Zn^2+^ to catalyze the cleavage of the substrate strand of Zn^2+^‐dependent DNAzyme, resulting in the release of anti‐miR‐21 and DOX after internalization by HeLa cells via endocytosis. These Zn^2+^‐dependent DNAzyme‐functionalized SNPs achieved drug delivery in a controlled manner and intracellular synergistic therapy.

Another example, Yu et al. functionalized the hollow mesoporous magnesium silicate nanoparticles (HMMSNs) with the complex of Mg^2+^‐dependent DNAzyme and its substrate (1‐RNA) as capping units to “lock” and “unlock” DOX (Figure 3c).^[^ ^138^ ^]^ In mild acidic conditions, the Mg—O bond of HMMSNs easily broke up to generate Mg^2+^ and further activated the Mg^2+^‐dependent DNAzyme to cleave the substrate 1‐RNA and accelerated the releasing of loaded DOX, of which releasing percentage reaches 60% at pH 5.5. Also Mg^2+^‐dependent DNAzyme‐decorated HMMSNs achieved a high anticancer effect, reflected by the tumor‐suppression efficiency up to 68.75%. More importantly, HMMSNs enabled biodegradation, and the biodegradable products exhibited neglectable cytotoxicity even at the high concentration of 400 μg mL^−1^ (Figure 3d). All results indicate that the Mg^2+^‐dependent DNAzyme‐functionalized HMMSNs can be used as relatively safe nanocarriers for the delivery of chemotherapeutics and hold highly promising potential for further clinical translations.

### 3.4 ASO‐Decorated SNPs

ASO is a synthesized single‐stranded RNA/DNA that can interfere with the translation of target complementary mRNAs by Watson–Crick base pairing rules.^[^ ^139^ ^]^ This appears via two main mechanisms. For the first one, ASO hybridizes with its target mRNA, and then the formed DNA–RNA heteroduplex can trigger endonuclease RNase H or argonaute 2‐mediated mRNA selective cleavage.^[^ ^140^ ^]^ For the second one, the DNA–RNA heteroduplex can prevent ribosome from binding mRNA to block protein synthesis.^[^ ^141^ ^]^ Especially, ASO designed to bind microRNAs (miRNAs) can increase protein expression by preventing interactions with mRNAs and miRNAs, which would reduce post‐translational protein expression.^[^ ^142^ ^]^ Similarly, ASO can interfere with the interaction between long noncoding RNAs and chromatin to increase gene expression.^[^ ^143^ ^]^

#### 3.4.1 Cancer Gene Therapy

By targeting oncogenes and interfering with their expression, ASOs are used as potential candidates for cancer gene therapy. However, naked ASOs are extremely unstable in serum due to enzymatic degradation and poor cell‐membrane permeability, so as to restrict their wide application. Hence, a number of ASOs have been conjugated onto the surface of SNPs as vehicles for efficient delivery of ASOs to a tumor site and enhanced cellular internalization. To date, ASO‐modified SNPs have been developed for ASO‐based gene therapy from the following respects: promoting apoptosis of cancer cells, inhibiting tumor metastasis, reversing chemotherapy resistance, and synergizing with PDT for cancer therapy (**Table** **3**).

**Table 3:** ASO‐decorated SNPs for gene therapy

Functions	Targets of ASOs	Spherical nanoparticles	Ref.
Promoting apoptosis of cancer cells	Survivin mRNA	MSNs	[46]
	miR‐21	MSNs, AuNPs	[58, 137]
	miR‐155	AuNPs	[147a]
	hTERT mRNA	AuNPs	[147b]
Inhibiting tumor metastasis	MALAT1 IncRNA	AuNPs	[152]
	Mutant Kras mRNA	AuNPs	[154]
Reversing chemotherapy resistance	P‐gp mRNA	Micelles	[156]
	Bcl‐2 mRNA	Micelles	[158]
Synergizing with PDT for cancer therapy	HIF‐1α mRNA	AuNPs	[160]

##### Promoting Apoptosis of Cancer Cells

Inducing programmed cell death through caspase‐dependent apoptosis is a rational approach for cancer therapy.^[^ ^144^ ^]^ A number of oncogenic miRNAs, such as miR‐21 and miR‐155, can down‐regulate the antiapoptotic genes, resulting in apoptosis resistance.^[^ ^145^ ^]^ In addition, several mRNAs, such as survivin and human telomerase reverse transcriptase catalytic subunit (hTERT), have been identified as antiapoptotic oncogenes.^[^ ^146^ ^]^ Blocking or down‐regulating the expression of these oncogenes by ASOs can inhibit cell growth and promote cell apoptosis, thereby reversing the malignant phenotype of tumor cells. Also the ASOs targeting these oncogenes have been grafted on the surfaces of intelligent SNPs, including MSNs,^[^ ^46^, ^58^ ^]^ AuNPs,^[^ ^137^, ^147^ ^]^ and demonstrated to achieve significant antitumor efficacy.

##### Inhibiting Tumor Metastasis

Metastasis is the major cause of poor prognosis in patients with cancer, and several metastasis‐related genes have been potential targets for cancer gene therapy. Metastasis‐associated lung adenocarcinoma transcript 1 (MALAT1) is a highly conserved nuclear noncoding RNA overexpressed in many malignant tumor types, including lung cancer,^[^ ^148^ ^]^ pancreatic cancer,^[^ ^149^ ^]^ colorectal cancer,^[^ ^150^ ^]^ etc. It was first identified that MALAT1 was associated with the formation of lung metastatic foci so as to promote tumor metastasis.^[^ ^151^ ^]^ Gong et al. constructed ASO of MALAT1 and nucleus‐targeting TAT peptide cofunctionalized AuNPs (ASO‐Au‐TAT NPs) for malignant lung cancer gene therapy.^[^ ^152^ ^]^ The ASO‐Au‐TAT NPs enhanced ASO uptake by A549 cells and then could efficiently transport ASO across the nuclear pore into the nucleus. Also the excellent suppressed cancer metastasis ability of ASO‐Au‐TAT NPs was demonstrated in both in vitro and in vivo experiments. In addition, Bao^[^ ^154^ ^]^ and co‐workers conjugated the Cy3‐labeled ASO designed to bind mutant *Kras* mRNA,^[^ ^153^ ^]^ a well‐known driver oncogene in human cancers, onto the surface of AuNPs to form *Kras* mRNA‐responsive gold nanobeacons. The ASO‐modified nanoplatform could not only reduce the metastasis of gastric tumors to the lung via inhibiting *Kras* mRNA but also detect the expression of a mutant *Kras* gene based on selective fluorescence recovery of Cy3.

##### Reversing Chemotherapy Resistance

**Figure 4:**
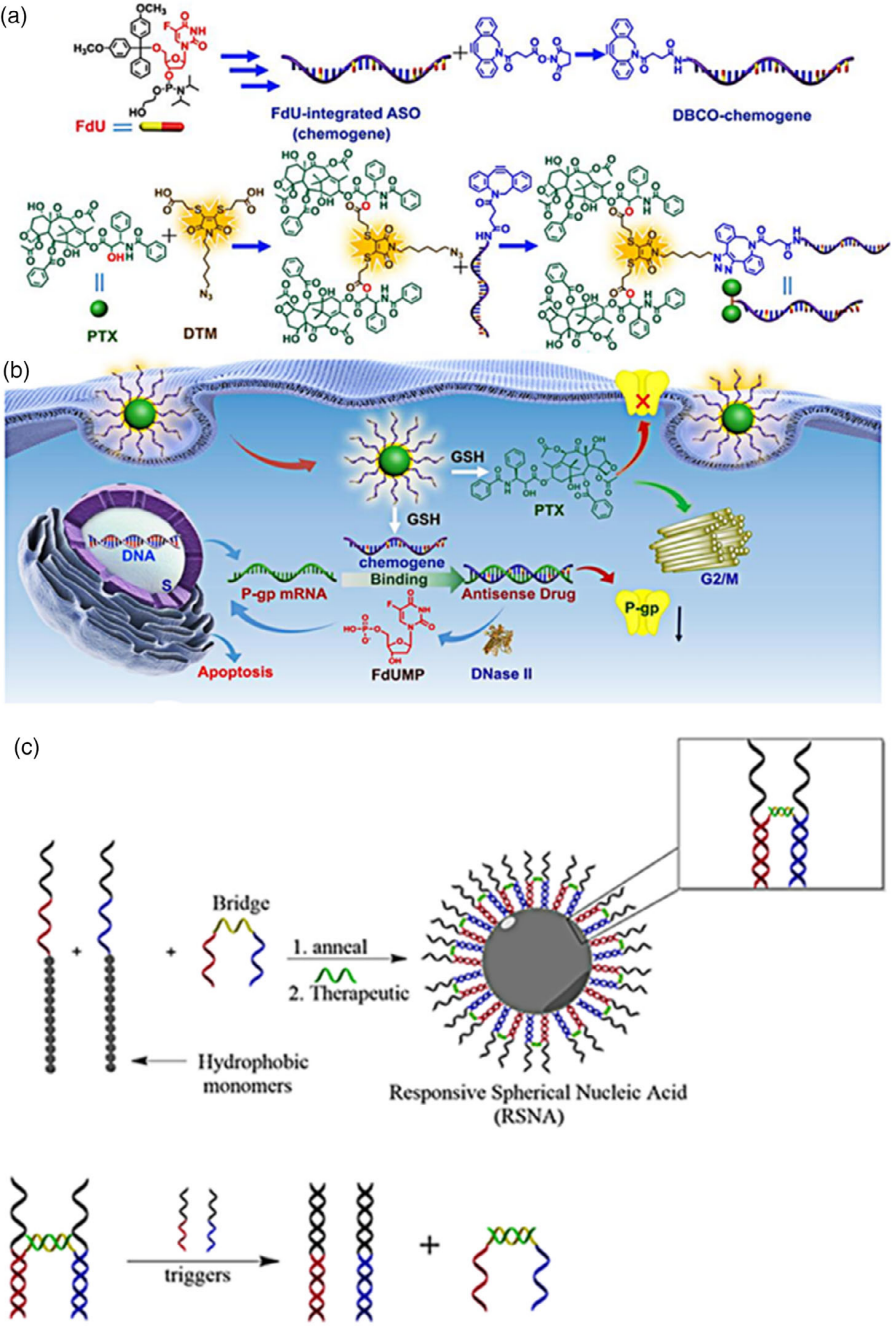
a) Schematic illustration for the synthesized route of PTX–(FdU‐integrated ASO) conjugates. b) The PTX–(FdU‐integrated ASO) assembled micelles for reversing the sensitivity of drug‐resistant tumor cells to chemotherapeutic drugs. a,b) Reproduced with permission.^[^ ^156^ ^]^ Copyright 2020, Wiley‐VCH. c) Schematic illustration for the design and cargo release mechanism of a responsive ASO‐modified micellar nanostructure. Reproduced with permission.^[^ ^161^ ^]^ Copyright 2019, American Chemical Society.

Resistance to chemotherapeutic drugs is the major obstacle for chemotherapy in cancer research. Drug‐resistant tumor cells adaptively upregulate the expression of multidrug resistance proteins such as P‐glycoprotein (P‐gp), a typical chemotherapeutic drugs efflux pump to decrease intracellular drug accumulation, which greatly hinders the effectiveness of cancer chemotherapy.^[^ ^155^ ^]^ Therefore, Zhu et al. combined P‐gp inhibitor (anti‐P‐gp ASO) with chemotherapeutic drugs (paclitaxel [PTX] and floxuridine [FdU]) to restore the sensitivity of tumor cells to drugs (**Figure** **4**a).^[^ ^156^ ^]^ Concretely, two PTX molecules were linked using dithiomaleimide as a bridge and then conjugated with a presynthesized FdU‐integrated anti‐P‐gp ASO (chemogene). Finally, the formed amphiphilic PTX–chemogene conjugates self‐assembled into ASO‐decorated spherical micellular nanoparticles. The carrier‐free drug‐delivery vehicle could release PTX and chemogene in response to high GSH due to DTM bond. It was demonstrated to inhibit the growth of drug‐resistant cancer through effectively downregulating the expression of P‐gp mRNA and subsequently releasing the FdU for DNase II degradation to induce cell apoptosis (Figure 4b).

In addition, several antiapoptosis proteins such as B‐cell lymphoma 2 (Bcl‐2) family proteins, which are highly expressed in tumor cells, are responsible for chemotherapy resistance through blocking the transcription of proapoptotic proteins such as cytochrome c and caspase activation.^[^ ^157^ ^]^ Also RNA‐inference mediated silencing of Bcl‐2 can mitigate antiapoptotic cellular defense so as to sensitize tumor cells to chemotherapeutic drugs. For example, Mou et al. synthesized anti‐Bcl‐2 ASO‐decorated micelles for curing drug‐resistant cancer.^[^ ^158^ ^]^ Specifically, the anti‐Bcl‐2 ASO integrated with FdU was conjugated with copolymer PEG‐*b*‐PCL and then self‐assemble to form a micellar spherical nanostructure. Results demonstrated that the FdU‐integrated ASO‐functionalized micelles downregulated the expression of Bcl‐2 proteins to effectively reverse drug resistance and significantly inhibited tumor growth in both subcutaneous and orthotopic drug‐resistant BEL‐7402 tumors‐bearing mouse models.

##### Synergizing with PDT for Cancer Therapy

PDT is a noninvasive cancer treatment that relies on the consumption of oxygen to produce cytotoxic reactive oxygen species. As a result, PDT can exacerbate hypoxia in tumors and then further stimulate upregulation of HIF‐1α, which mediates a variety of downstream genes to promote tumor development.^[^ ^159^ ^]^ Therefore, Huang et al. constructed a hypoxia‐triggered drug delivery system to achieve synergistic cancer PDT/anti‐HIF‐1α gene therapy.^[^ ^160^ ^]^ Concretely, a double‐strand AS1411/RX‐0047 (anti‐HIF‐1α ASO) hybridization complex was attached to the surface of β‐CD‐coated AuNPs via a hypoxia‐induced cleaved Azo bridge. The obtained ASO‐decorated AuNPs could deliver and release anti‐HIF‐1α ASO under the exacerbated hypoxia environment that was induced by PDT based on photosensitizer TMPyP_4_, resulting in enhanced the tumor‐killing efficacy of PDT.

#### 3.4.2 Controlling the Release of Chemotherapeutic or Nucleic‐Acid‐Based Drugs

Due to its selectivity and specificity, ASO targeting a cytoplasmic genetic marker has been utilized to design stimuli‐responsive SNPs for conditional delivery of chemotherapeutic drugs and/or nucleic‐acid‐based drugs. For example, Zhang et al. constructed a DNA hybrid‐capped mesoporous silica‐coated quantum (MSQDs) for controlling DOX release.^[^ ^58^ ^]^ The DNA hybrid, serving as the gate to prevent leaking of DOX, is composed of AS1411 and anti‐miR‐21, which was located at both ends of the AS1411 aptamer. The anti‐miR‐21 partially hybridized with anchor DNA on the MSQDs, and its target overexpressed endogenous miR‐21 functions as an exclusive key to competitively hybridize with anti‐miR‐21 of the DNA hybrid, resulting in the release of DOX from MSQDs. More importantly, it was demonstrated that anti‐miR‐21 ASO as the endogenous miR‐21 inhibitor contributed to enhanced efficacy of chemotherapy.

In addition, a two triggers‐responsive ASO‐modified micellar nanostructure was designed by Fakih et al. (Figure 4c).^[^ ^161^ ^]^ The micelles were prepared using three components: two “pillar” DNA‐polymer amphiphilic strands and a “bridge” strand. The two “pillar” strands were designed for responding to two specific cytoplasmic genetic markers (e.g., miRNAs) that are overexpressed in cancer cells. The “bridge” strand was designed to partially hybridize with the two “pillar” strands to form responsive FSNPs, and its middle section can load nucleic‐acid drugs (green), here using luciferase ASO as an example, through complementary base pairing. Only meeting two genetic markers of interest (e.g., miRNAs) inside tumor cells, the “bridge” strand disassembled from MSNAs via strand displacement to further release nucleic‐acid drugs. The designed dual‐stimuli responsive ASO‐modified SNPs contribute in improving the accuracy and specificity of responsiveness, allowing for minimized off‐target effects on cells.

### 3.5 CpG ODN‐Decorated SNPs

Evading immune destruction was implicated in the development of cancer,^[^ ^162^ ^]^ so stimulating immune activation becomes a promising strategy for cancer therapy. ODN with unmethylated deoxycytidyl‐deoxyguanosine (CpG) dinucleotides (CpG ODN), one type of synthetic immunostimulatory oligonucleotide, can be recognized by toll‐like receptor 9 (TLR9) in immune cells, such as B cells, plasmacytoid dendritic cells (pDC), macrophages, and MDSCs,^[^ ^163^ ^]^ and then stimulate these cells activation to secret type I IFN and cytokines.^[15b]^ CpG ODNs are generally categorized into three classes with distinct immunostimulatory activities, including A‐class, B‐class, and C‐class. A‐class CpG ODNs (CpG A, also termed D‐type) contains a CpG‐containing palindrome with a phosphodiester (PO) backbone surrounded by poly(G) sequences with a phosphorothioate backbone. CpG‐A strongly induces pDCs to produce IFN‐α but poorly activates B‐cell proliferation. In contrast, B‐class CpG ODNs (CpG‐B, also termed K‐type) comprise hexamer CpG motifs with a full phosphorothioate backbone and strongly activates B‐cell proliferation and pDC maturation, but moderately induces production of IFN‐α by pDC. C‐class CpG ODNs show intermediate responses between A‐ and B‐classes.^[^ ^164^ ^]^

#### 3.5.1 Enhancing Immunostimulation Activity of CpG ODNs

CpG ODNs have been popularly used as potent adjuvants for the treatment of cancers. However, free CpG ODNs are difficult to attain high cell‐uptake efficiency and easy to be degraded by nucleases. Thus, a variety of SNPs have been developed as vehicles to deliver CpG ODNs. For example, Lin et al. conjugated CpG ODNs onto the surface of AuNPs and demonstrated that nanoparticle conjugation enhanced the delivery and immunostimulation activity of CpG ODNs to macrophages and subsequently significantly inhibited tumor growth.^[^ ^165^ ^]^

Undoubtedly, the immune activation of CpG ODN‐modified SNPs is positively related to the surface density of CpG ODN.^[^ ^48^ ^]^ Interestingly, Pallares et al. found that just a 5% rate of CpG to total ODN (high density) on the surface of AuNPs was sufficient to induce maximal immune‐activation responses of macrophages, implying ligand shell tunability of CpG ODN‐modified SNPs.^[^ ^166^ ^]^ In addition, the sizes of SNPs can influence their immunostimulatory effects. Yue et al. prepared two different sizes (13 and 50 nm) of spherical AuNPs cores with the same CpG surface density.^[^ ^167^ ^]^ They found 50 nm spherical AuNPs exhibited lower TLR9 targeting specificity than 13 nm spherical AuNPs, but higher cellular uptake and immune responses, resulting from target TLR9, as well as nontarget activation, such as TLRs 7 and 8, which would cause autoimmune disorders. This result highlights both structural parameters and ligand densities need to be considered when designing SNPs for reducing adverse side effects.

Although CpG ODNs‐modified SNPs can exert immunostimulatory capabilities, single CpG‐ODNs, such as CpG‐B, just moderately induces production of IFN‐α by pDC, and thus is weak for promoting tumor‐bearing mice survival.^[^ ^168^ ^]^ Therefore, Mirkin and co‐workers constructed immunomodulatory SNAs by incorporating both CpG‐B and CpG‐A ODNs on the surface of liposomes.^[^ ^169^ ^]^ The (dual‐CpG)‐functionalized liposomal SNAs exhibited higher cellular uptake than single‐component‐functionalized ones, as well as fivefold higher codelivery efficiency of CpG‐A and CpG‐B than mixtures of the two linear ODN counterparts, leading to synergistic DC activation. In addition, as the preferred intracellular organelles of CpG‐A and CpG‐B for TLR9 activation are different, i.e., early endosome and late endosome, respectively, and SNAs are retained in late endosome for longer periods of time than early endosomes, controlling rationally CpG‐A/B ratios is a good strategy to maximum DC maturation of the (dual‐CpG)‐functionalized liposomes.

#### 3.5.2 Functioning as Adjuvants of Cancer Vaccines

CpG ODNs, as potent TLR‐9 agonists with negligible toxicity, enable dendritic cell activation to promote antigen presentation. Therefore, codelivery of CpG ODNs as adjuvants and antigens has been widely explored in promoting the effectiveness of cancer vaccines. For example, Jin et al. constructed a vaccine formulation containing both CpG motifs and ovalbumin1 peptides, which is commonly used as a model antigen.^[^ ^170^ ^]^ The vaccine formulation was prepared through self‐assembly of three segments, including a 12‐nucleotide long DNA–lipid carrier, a 12‐nucleotide extended CpG sequence (eCpG), and a peptide nucleic acid–ovalbumin1 peptide conjugate (pOVA). The extended 12 nucleotides of eCpG and peptide nucleic acid sequence of pOVA were complimentary to the lipid‐DNA. They demonstrated that the vaccine formulation significantly enhanced antitumor and antimetastasis effects against melanoma. Functional genipin crosslinked ovalbumin (OVA) nanoparticles were reported to monitor the process of antigen delivery in real‐time based on the fluorescence of genipin.^[^ ^171^ ^]^ Accordingly, Dong et al. decorated the OVA nanoparticles with TAMRA‐labeled CpG ODNs.^[^ ^172^ ^]^ The fluorescent nanovaccine not only achieves antigen presentation and DCs activation for antitumor immunotherapy but also enables precise and visible codelivery of OVA and CpG ODNs through the dual‐fluorescence imaging method. These results indicate that visible codelivery of adjuvant and antigen strategy is worth to be considered when designing multifunctional cancer vaccines.

Apart from OVA1, tumor‐associated antigens such as human melanoma‐specific antigen gp100, melanoma antigen Trp2, human papillomavirus‐16 oncoprotein E6 antigen, carcinoma antigen peptide AH1, prostate‐specific antigen PSA_65−73_, and prostate‐specific membrane antigen PSMA_634−642_, as well as tumor proteins of the whole‐cell lysate from mouse H22 hepatoma cells and 4T1 breast tumor cells, have been reported to combine with CpG ODNs for eliciting an antitumor immune response.^[^ ^173^ ^]^ Recently, Wang and co‐workers constructed three CpG ODN‐functionalized liposomal SNAs nanostructures incorporated with antigens through different approaches, i.e., directly encapsulating into the core of liposomal SNAs, attaching onto the surface of liposomal SNAs by a hydrophobic anchor molecule Chol, and a conjugated peptide nucleic‐acid sequence capable of hybridizing with CpG ODNs, namely SNAs E, SNAs A, and SNAs H, respectively.^[173f]^ Using E6 and OVA1 as the antigen model separately, they found that SNAs H showed the best antitumor immunotherapeutic outcomes in relevant TC‐1 tumor‐bearing and OVA1‐tumor‐bearing mouse models, respectively, suggesting the antitumor immune response of SNAs incorporated with CpG ODNs and antigens was highly SNA structure‐dependent, and thus emphasizes the potential of rationally designed SNAs in systematically optimizing vaccine effectiveness for cancer.

For better DCs targeting, Lai et al. constructed mannose‐modified liposomes for codelivery of CpG ODN 1826 and melanoma‐specific antigen TRP2180‐188 peptide.^[^ ^174^ ^]^ As expected, the liposomal vaccine (M/CpG‐ODN‐TRP2‐Lipo) was efficiently taken up by DCs and displayed an enhanced antitumor‐specific immune response. Furthermore, a similar liposomal vaccine coated with mannose and CpG ODN for the delivery of B16 melanoma whole‐cell lysates (M/CpG‐ODN‐B16‐Lipo) was prepared. Interestingly, results demonstrated that M/CpG‐ODN‐TRP2‐Lipo induced stronger antitumor immune responses and improved survival of tumor‐bearing mice compared with M/CpG‐ODN‐B16‐Lipo, probably due to the existence of the nonantigenic proteins from cell lysates.

Photothermal therapy is well‐known to induce cellular hyperthermia‐based necrosis and subsequently release tumor‐associated antigens to trigger specific antitumor immunity. However, the TME is immunosuppressive. Thus, Li et al. developed an endogenous vaccine based on IR‐7‐loaded CpG ODN‐modified liposomes for synergistic photothermal and immunotherapy.^[^ ^175^ ^]^ The combined photothermal immunotherapy enables regulation of tumor immunosuppressive microenvironment and confers an advantage over surgical resection in tumor eradication and tumor metastasis inhibition.

## 4 Conclusions and Perspectives

Advances in the discovery of a serial of functional molecules have sparked interest in the development of various decorative nanoplatforms for applications in cancer therapy. Based on their molecular recognition, responsiveness, catalytic activities, and therapeutic potential, FNAs are promising macromolecules to decorate SNPs toward the ultimate goal of improving therapeutic efficacy while minimizing systemic side effects, as demonstrated by the selected examples described in this review. To date, FNA‐modified SNPs have been implemented to enhance targeted cancer therapy, control the release of drugs, overcome multidrug resistance, activate the antitumor immune response, and improve the permeability to interstitial transport barriers. More importantly, multifunctional FNAs exist, and multiple types of FNAs can be decorated on the surface of the same spherical core simultaneously, which afford more intelligent and additional properties for SNPs toward smart cancer therapy.

Albeit with the exciting progress, several challenges must be addressed. First, although numbers of FNAs have been discovered, the majority of studies tend to use several classical FNAs, such as anti‐miR‐21 and AS1411, suggesting that more excellent FNAs are waiting to be discovered. ASOs could be designed aiming at the sequence of the desired target gene via the Watson–Crick base‐pairing rule, but aptamer is hard to be designed due to the fact that the accurate recognition mechanism of aptamer with its target is unclear. In general, the discovery of aptamer is mainly dependent on the SELEX technology, and a large number of new aptamers targeting important cancer‐related proteins or cells are expected to be identified through the high throughput SELEX process. In addition, compared with conventional protein or cell SELEX, in vivo SELEX should get more attention for the generated aptamers definitely not binding to blood cell‐surface proteins.

Second, the cellular internalization for the FNAs, as the main functional component of FSNPs, is a key step for achieving optimal therapy. In general, FNAs are taken up by cells through the endocytic pathway.^[^ ^176^ ^]^ Although the endocytosis mechanism of other FNAs is uncertain, the aptamer has been well studied and clarified as clathrin‐ and caveolae‐mediated endocytosis, macropinocytosis, and phagocytosis.^[^ ^177^ ^]^ Nevertheless, the cellular internalization ability of FNAs is low because of strong electrostatic repulsion between both negatively charged FNAs and cell membranes. Mirkin and co‐workers first introduced SNAs, spherical nanostructures with densely functionalized and highly oriented nucleic acids covalently attached to their surfaces. They demonstrated that FNAs decorated onto the surface of SNAs were rapidly internalized into different cell lines and showed more than 90% cellular uptake without cationic vectors, significantly enhancing intracellular delivery of FNAs and decreasing side effects.^[^ ^178^ ^]^ In addition, both the surface density and type of FNAs affected their uptake by target cells. More exacted mechanism of the cellular internalization for FNAs remained to be explored, and more “smart” strategies need to be designed to improve the rate of cellular uptake. After cell internalization, FNAs traffic through early endosomes, and most of FNAs are ultimately delivered to late endosomes and lysosomes for degradation and hard to escape endosomes. If FNAs stay in transport vesicles all the way, lysosomes can completely metabolize FNAs using hydrolytic and enzymatic reactions, resulting in the limited therapeutic utility of FNAs. Although SNAs can enhance cellular uptake of the FNAs via a caveolae/lipid‐raft‐dependent pathway,^[^ ^179^ ^]^ endosomal escape is still an important problem for SNAs. For improving the endosomal escape of FSNPs, several kinds of researches have currently been used, such as PDA surface coating and cationic cell‐penetrating peptide KALA complexing, which are probably explained as the “proton sponge” effect and decreasing membrane stability, respectively. In addition, cationic lipid 1,2‐dioleoyl‐*sn*‐glycero‐3‐phosphoethanolamine (DOPE), usually used as a component of liposomes, can enhance the translocation of FNAs from endosomes to the cytosol.^[^ ^180^ ^]^ Thus, conjugation of FNAs with SNPs using endosomal escaping polymers or cell‐penetrating peptides as linkers is worth studying.

Finally, FSNPs are composed of three functional units, including FNAs, SNPs, and links, each of which function is no single yet. For instance, several FNAs, such as aptamers, apart from targeting special proteins/cells, have been reported for the loading of small drugs and siRNA. However, the drug‐loading efficiency of the aptamer is relatively limited due to its small size compared with SNPs. Thus, the aim of the combination of multiple functional units is to make the best use of the advantages and bypass the disadvantages, but not pile up complicated nanoplatform, which is unbeneficial for clinical translation of FSNPs.

## Conflict of Interest

The authors declare no conflict of interest.
